# Glipizide, an antidiabetic drug, suppresses tumor growth and metastasis by inhibiting angiogenesis

**DOI:** 10.18632/oncotarget.2483

**Published:** 2014-09-16

**Authors:** Cuiling Qi, Qin Zhou, Bin Li, Yang Yang, Liu Cao, Yuxiang Ye, Jiangchao Li, Yi Ding, Huiping Wang, Jintao Wang, Xiaodong He, Qianqian Zhang, Tian Lan, Lee Kenneth Ka Ho, Weidong Li, Xiaoyu Song, Jia Zhou, Xuesong Yang, Lijing Wang

**Affiliations:** ^1^ Vascular Biology Research Institute, Guangdong Pharmaceutical University, Guangzhou, China; ^2^ Key Laboratory for Regenerative Medicine of the Ministry of Education, Division of Histology & Embryology, Medical College, Jinan University, Guangzhou, China; ^3^ Key Laboratory for Regenerative Medicine of the Ministry of Education, School of Biomedical Sciences, Chinese University of Hong Kong, Shatin, Hong Kong, China; ^4^ Key Laboratory of Medical Cell Biology, China Medical University, He Ping District, Shen Yang City, Liao Ning Province, China; ^5^ Chemical Biology Program, Department of Pharmacology and Toxicology, University of Texas Medical Branch, Galveston, TX, United States

**Keywords:** glipizide, anticancer, metastasis, tumor angiogenesis, natriuretic peptide receptor A

## Abstract

Angiogenesis is involved in the development, progression and metastasis of various human cancers. Herein, we report the discovery of glipizide, a widely used drug for type 2 diabetes mellitus, as a promising anticancer agent through the inhibition of tumor angiogenesis. By high-throughput screening (HTS) of an FDA approved drug library utilizing our *in vivo* chick embryo chorioallantoic membrane (CAM) and yolk sac membrane (YSM) models, glipizide has been identified to significantly inhibit blood vessel formation and development. Moreover, glipizide was found to suppress tumor angiogenesis, tumor growth and metastasis using xenograft tumor and MMTV-PyMT transgenic mouse models. We further revealed that the anticancer capability of glipizide is not attributed to its antiproliferative effects, which are not significant against various human cancer cell lines. To investigate whether its anticancer efficacy is associated with the glucose level alteration induced by glipizide application, glimepiride, another medium to long-acting sulfonylurea antidiabetic drug in the same class, was employed for the comparison studies in the same fashion. Interestingly, glimepiride has demonstrated no significant impact on the tumor growth and metastasis, indicating that the anticancer effects of glipizide is not ascribed to its antidiabetic properties. Furthermore, glipizide suppresses endothelial cell migration and the formation of tubular structures, thereby inhibiting angiogenesis by up-regulating the expression of natriuretic peptide receptor A. These findings uncover a novel mechanism of glipizide as a potential cancer therapy, and also for the first time, provide direct evidence to support that treatment with glipizide may reduce the cancer risk for diabetic patients.

## INTRODUCTION

Angiogenesis is a cellular process that involves the sprouting of capillaries and configuration of the neovasculature from existing blood vessels [1,2]. Physiological angiogenesis occurs mainly during embryogenesis to accommodate the requirements of development and for this reason, only 0.01% the epithelial cells (ECs) undergo cell division in adults [1]. Nevertheless, angiogenesis plays a critical role in a variety of pathological conditions such as rheumatoid arthritis, diabetic retinopathy, inflammation, stroke and carcinogenesis [2,3]. Growth of the solid tumors requires a corresponding expansion of the vascular networks to maintain the increasing demand for blood supply. When this supply is insufficient, it leads to tumor cell necrosis and apoptosis or metastasis - depending on the aggressiveness of the neoplastic cells and their microenvironment [3]. Therefore, developing novel agents that are capable of inhibiting tumor angiogenesis represents a new paradigm in cancer prevention and treatment. Chick embryo chorioallantoic membrane (CAM) and yolk sac membrane (YSM) as *in vivo* models of angiogenesis were established and commonly utilized to facilitate the study of tumor angiogenesis and development of anti-angiogenic or pro-angiogenic agents [4,5]. We envisioned that high-throughput screening (HTS) of commonly used medicines with our *in vivo* CAM and YSM assays may lead to the discovery of novel anticancer therapeutics aimed at tumor angiogenesis.

Glipizide is a widely used drug in the treatment of type II diabetes since the 1950s because of its ability to stimulate insulin secretion from β-cells [6-8]. Recent studies have shown that diabetic patients have higher risks of developing colorectal, liver, pancreatic and prostate cancers [9-14]. Intriguingly, epidemic study has revealed that long-term use of some antidiabetic drugs such as gliclazide and glibenclamide alone or in combination with glipizide may result in a reduced risk of developing cancer in a dose-dependent manner from a cohort study of 6103 type-2 diabetic patients [15]. Nevertheless, it remains unclear how this class of antidiabetic drugs is associate with the decreased cancer risk in the diabetic patients with these treatments. The findings disclosed in our study provide the direct evidence to support such outcomes of glipizide in cancer prevention associated with the inhibition of angiogenesis.

Natriuretic peptides (NPs) belong to a family of polypeptide hormones that contains three isoforms: atrial natriuretic peptide (ANP), B-type natriuretic peptide (BNP), and C-type natriuretic peptide (CNP). These NPs are produced by the heart, vasculature and kidney. They play an important role in cardiovascular homeostasis, which is mediated by their receptors, natriuretic peptide receptors A and B (NPRA and NPRB) through the cytoplasmic guanylyl cyclase domains. NPRA is strongly expressed in the vasculature, kidneys and adrenal glands while NPRB is expressed in the brain. NPRA is also expressed in tumor cells (such as lung and prostate cancer cells) and inhibition of NPRA has been reported to inhibit tumor growth by suppressing cell proliferation [16-18]. Furthermore, activation of NPRA signaling could result in tumor growth by inducing stem cell recruitment and angiogenesis [19].

In this study, by screening of an FDA approved drug library utilizing our *in vivo* CAM and YSM models, glipizide has been identified to significantly inhibit blood vessel formation and tumor development. Here we report that glipizide inhibits tumor growth and metastasis in 4T1 transplanted tumor and spontaneous breast cancer in MMTV-PyMT transgenic mice. Further studies suggest that glipizide inhibits tumor-induced angiogenesis through up-regulation of NPRA, thereby suppressing tumor growth and metastasis.

## RESULTS

### Glipizide inhibits angiogenesis in CAM and YSM models

**Figure 1:**
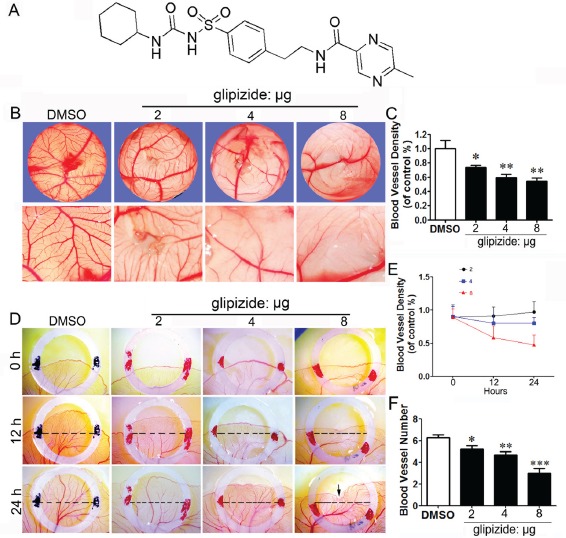
Glipizide inhibits angiogenesis in embryonic CAM and YSM assays Chick CAM and YSM assays were used to determine whether glipizide can inhibit angiogenesis to suppress tumor growth. (A) Chemical structure of glipizide. (B) The upper panels show that the blood vessel plexus of 11-day-old chick embryos following treatment with 2, 4 and 8 μg of glipizide or DMSO. The lower panels show the appearance of blood vessel plexus at higher magnification as indicated by white dotted squares in upper panels. (C) The bar chart shows the relative blood vessel density on CAM following glipizide and DMSO treatment. (D) The entire egg content was transferred into a sterile petri dish after two-day incubation. The upper panels show the appearance of the blood vessel plexus at the start of experiment (0 h) for control and 2, 4 and 8 μg of glipizide. The middle and lower panels show the appearance of blood vessel plexus after 12 h and 24 h incubation, respectively. (E) Statistical chart shows the blood vessel density for control and glipizide treatment group. (F) Bar chart compares the number of blood vessels on YSM between control and glipizide treatment group. * p < 0.05; ** p < 0.01.

The high-throughput screening of an FDA-approved drug library of 480 compounds was carried out utilizing our CAM and YSM assays, aiming at the discovery of potentially new antiangiogenic molecules. In our initial effort using the CAM approach, 27 potential antiangiogenic drugs have been identified, and subjected to the investigation of effects inhibiting angiogenesis using the YSM assay. Consequently, one compound was narrowed down out of the 27 compounds (Supplemental Table S1), leading to the discovery of glipizide (Its chemical structure shown in Figure 1A) as the most effective drug in inhibiting angiogenesis.

We further validated the effect of glipizide on angiogenesis by simply administrating glipizide on the vascular plexus of CAM and YSM. Chick embryos were treated with 2, 4 and 8 μg of glipizide or DMSO (control) for 48 h and then photographed (Figure 1B). We found that glipizide treatment led to a significant reduction in the density of CAM blood vessel plexus as compared with the control (Figure 1C). The YSM model was used to further confirm that glipizide could inhibit angiogenesis. Unshelled fertilized eggs were incubated inside sterile petri dishes for 9 days and then treated with glipizide (2, 4 and 8 μg) or DMSO for 24 h within plastic rings (Figure 1D). The vascular beds on the egg yolk were photographed at 0, 12 and 24 h. The results revealed that extension of the blood vessel plexus was significantly inhibited by exposure to glipizide (Figure 1D). The blood vessel density was also significantly impaired by glipizide (Figures 1E and 1F). These data suggest that glipizide directly affects angiogenesis during the development of chick embryo.

### Glipizide suppresses 4T1 and B16F10 xenograft tumor growth and metastasis

**Figure 2:**
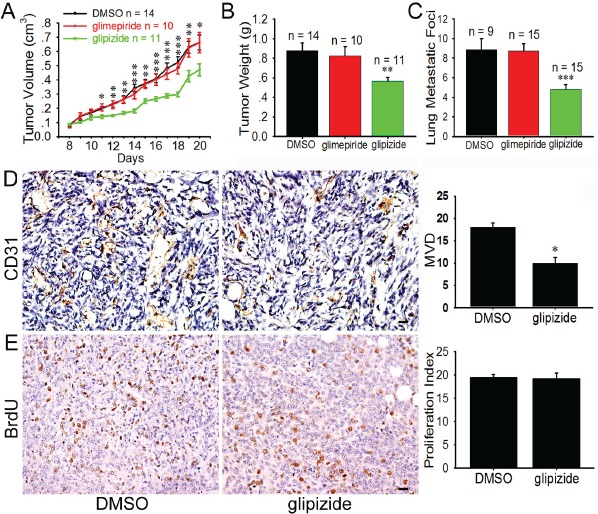
Glipizide inhibits angiogenesis, tumor growth and metastasis of breast cancer 4T1 cells 4T1 cells were injected into mammary mouse pad and when tumors were palpable on day 7, they were injected with glipizide (5 mg/kg), glimepiride (4 mg/kg) or DMSO (control) once every day. (A) The volume of the breast tumors was measured every day and revealed that the tumor volume significantly decreased after glipizide treatment. (B) Tumors were weighed on 20 days after inoculation. There was significant difference between the weights of glipizide and glimepiride or DMSO treated tumors. (C) Incidence of lung metastasis enumerating from the breast tumors after glipizide, glimepiride and DMSO treatments. (D) Immunohistological staining against CD31 was performed on tumor tissues sections. The staining shows the microvascular density was decreased in the glipizide treatment group. (E) Glipizide displayed no significant effect on tumor cell proliferation. Abbreviation: MVD, microvascular density. Results are given as mean ± S.D. Values of three independent experiments (A-C) or at least five randomly selected sections per animal (D). Scale bars = 20 μm in D and E. * *p < 0.05*; ** *p < 0.01*; *** *p < 0.001*.

Given that glipizide is capable of inhibiting angiogenesis in the chick embryo, we speculated that glipizide may also suppress tumor-induced angiogenesis, thereby mitigating tumor growth and metastasis. To this end, we employed a mouse xenograft model with mouse breast cancer 4T1 cells. Glipizide (5 mg/kg) and glimepiride (4 mg/kg) were administered daily for 14 days after subcutaneous inoculation of 4T1 cells into mouse mammary fat pad. Glipizide was found to significantly inhibit tumor growth (Figure 2A) and weight (Figure 2B). In contrast, glimepiride has no significant effect on the tumor growth. Compared with the DMSO treatment, the postprandial blood glucose levels of the mice treated with the glipizide and glimepiride significantly decreased 30 min later and returned to normal 12 h later (Supplemental Table S2). Glipizide treatment also significantly reduced lung metastasis when 4T1 cells were intravenously administered as compared with the DMSO or glimepiride group (Figure 2C). Furthermore, glipizide was also found capable of inhibiting B16F10 melanoma growth and metastasis (Supplemental Figure S1). In addition, glipizide abolishes the CD31 staining for MVD (Figure 2D). However, there were not significant effects on tumor cell proliferation in glipizide-treated tumor as compared with the control (Figure 2E). These results collectively suggest that glipizide inhibits the tumor growth and metastasis of malignant melanoma and breast cancer by impeding tumor-induced angiogenesis.

### Glipizide impedes spontaneous tumor growth and metastasis in MMTV-PyMT transgenic mice

**Figure 3:**
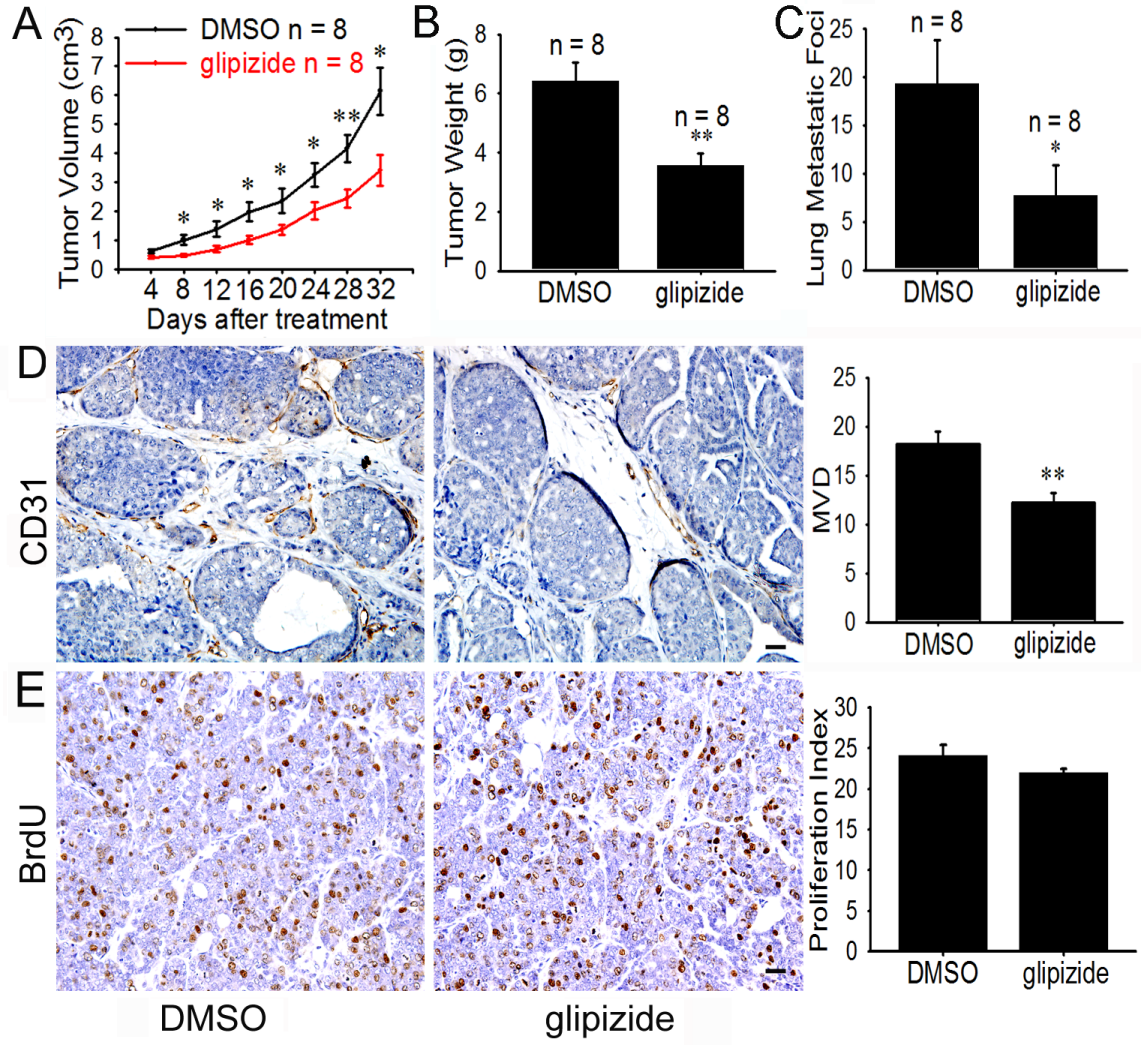
Glipizide suppresses angiogenesis, tumor growth and metastasis of spontaneous breast cancer (A) 9-week-old female MMTV-PyMT mice were allowed to spontaneously develop breast cancer and then given DMSO or glipizide (5 mg/kg) intraperitoneally, once every three days. The results show glipizide inhibits tumor growth. (B and C) After the mice were sacrificed, both the lungs and the breast tumor were harvested. The presence of metastatic foci on the lung surface was counted and the breast tumors weighed. The results show glipizide reduces the number of lung metastatic foci but has no effect on the weight of the tumors. (D and E) Immunohistological staining on tumor tissues sections revealed that glipizide inhibited tumor-induced angiogenesis, but not tumor cell proliferation. Abbreviation: MVD, microvascular density. Scale bars = 20 μm in D and E. * *p < 0.05*; ** *p < 0.01*.

The inhibitory effect of glipizide on tumor growth and metastasis was further investigated using the spontaneous murine model of breast cancer. MMTV-PyMT transgenic mice spontaneously develop widespread multifocal adenocarcinomas in the entire mammary epithelium which develop into palpable mammary tumors in 5 weeks and older mice. These tumors metastasize to the lung with 80-94% incidence [20]. To study the effect of glipizide on the spontaneous mammary tumors, we randomly selected eight MMTV-PyMT mice as the control and another eight for glipizide treatment (5 mg/kg). The tumor volumes were measured and calculated every 4 days. Comparison of the control and glipizide treatment group revealed that the tumor volume in glipizide-treated mice was significantly reduced (Figure 3A). Meanwhile, the blood glucose levels were significantly decreased at 30 min after the glipizide treatment (Supplemental Table S3). The mice were sacrificed and tumors harvested 20 days after the glipizide treatment. There was significant difference in the tumor weight between control and glipizide-treated mice (Figure 3B). Intriguingly, it was found that there were fewer metastasis sites on the lung surface of glipizide treatment mice (Figure 3C). Immunohistological staining for CD31 was performed on the tumor sections. The results demonstrated a reduction in microvessel density in glipizide-treated tumor as compared with the control (Figure 3D). However, glipizide had no significant effect on the BrdU staining for tumor cell proliferation (Figure 3E).

### Glipizide inhibits MCF-7 cell growth and angiogenesis on CAM

**Figure 4:**
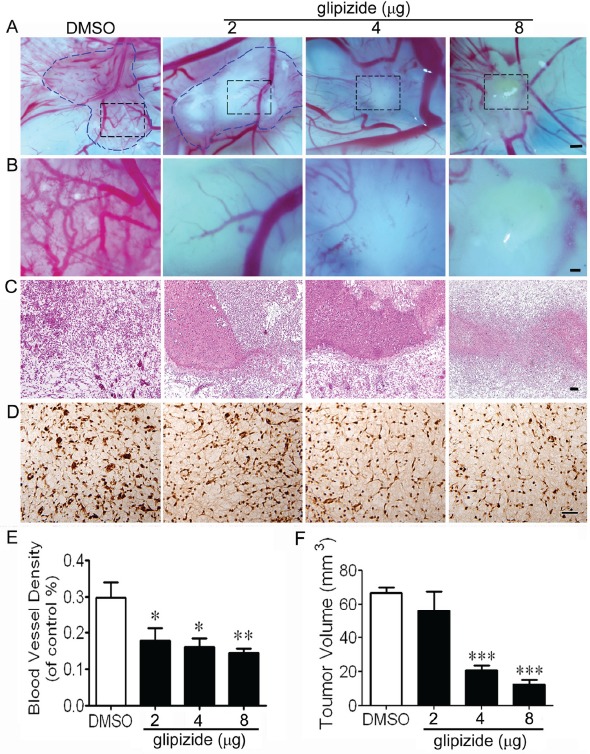
Glipizide suppresses breast cancer growth and angiogenesis MCF-7 breast cancer cells were xenografted onto the surface of 8-day chicken CAM. (A) Representative images of the cancer xenografts treated with DMSO or 2, 4 and 8 μg of glipizide. (B) Higher magnification of the black dotted squares in A. (C) Xenografts stained with H&E. (D) ISH staining against VEGFR-2 was performed and the staining shows the expression of VEGFR-2 during experimental breast cancer development. (E and F) Bar chart shows the differences in blood vessel density and tumor volume measurement of glipizide and DMSO treated xenografts on CAM. * *p < 0.05*; ** *p < 0.01*. Scale bars = 500 μm in A; 200 μm in B and 50 μm in C.

Correlation between tumor growth and angiogenesis was further investigated by introducing MCF-7 cells to the surface of 10-day old embryo CAM. The CAM model allows glipizide (2, 4 and 8 μg) to directly exert its effect on tumor angiogenesis (Figures 4A, 4B and 4E). We found tumor volume and microvascular density were significantly reduced following glipizide treatment in a dose-dependent manner as compared with control (Figures 4E and 4F). H&E staining revealed that in the control (DMSO) group there was no necrosis amongst the blood vessels. However, necrosis increased with decrease in blood vessel density following glipizide treatment (Figure 4C). Furthermore, we investigated the expression of VEGFR-2, which is specific in capillary endothelial cells. It was found that the expression of VEGFR-2 was gradually down-regulated in blood vessels with increasing the concentration of glipizide (Figure 4D). These results indicate that glipizide reduces breast cancer growth by inhibiting tumor-induced angiogenesis *in vivo*.

### Glipizide does not affect 293T, 4T1 and MCF-7 cell proliferation, but inhibits the HUVEC migration and tube formation

**Figure 5:**
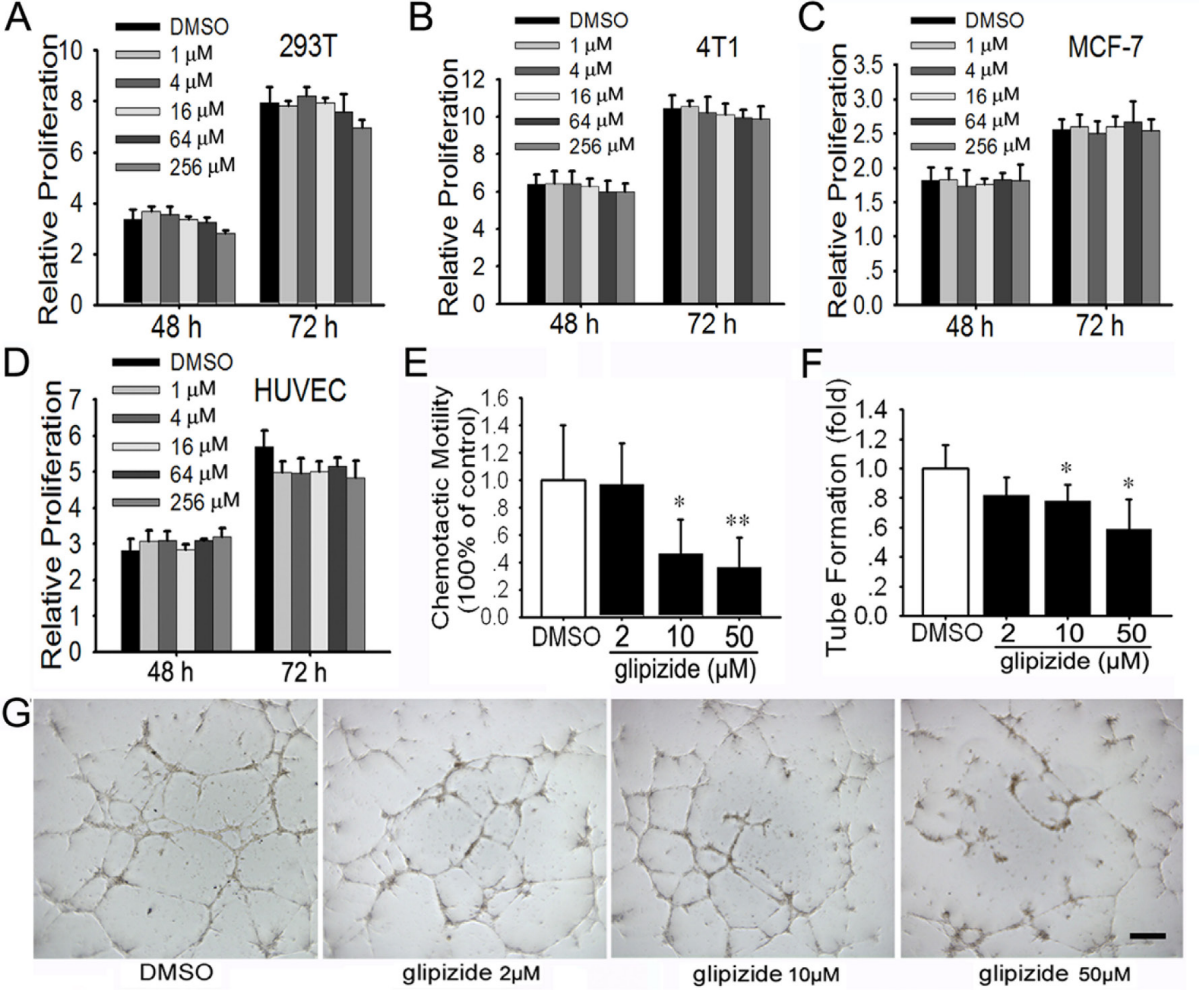
Glipizide shows no effects against cell proliferation, but inhibits endothelial cell migration and tube formation (A-C) MTT assay was performed on 293T (human kidney epithelium) (A), 4T1 (mouse breast cancer) (B), and MCF-7 (human breast cancer) cells (C) after treatment with 1 - 256 μM of glipizide for 48 h or 72 h. The results show no effect on cell proliferation and viability. (D) HUVEC cells were treated with 1 - 256 μM of glipizide for 48 or 72 h to establish the effect of glipizide on HUVEC proliferation. MTT assay shows 1 - 256 μM of glipizide does not suppress HUVEC proliferation. (E) The migration ability of HUVEC was evaluated using boyden chambers. HUVECs suspended various concentrations of glipizide were introduced into the upper chamber and allowed to migrate through the interposing filter for 12 h. The results show HUVECs migration was significantly reduced in the presence of 10 and 50 μM of glipizide. (F and G) HUVECs were cultured on matrigel in the presence of glipizide or DMSO for 5 h and the formation tubes were recorded (G). The length of the tubes was measured and difference between glipizide and DMSO treatment was statistically analyzed (F). Data are presented for at least three independent experiments. * p < 0.05, ** p < 0.01. Scale bars = 50 μm in G.

Now that glipizide inhibits breast cancer growth and metastasis *in vivo*, it is interesting to explore whether glipizide directly inhibits tumor cell proliferation. Hence, we evaluated the effects of glipizide on human kidney epithelial 293T, 4T1 and MCF-7 cell proliferation. These cells were treated with 1 – 256 μM of glipizide for 48 or 72 h. Cell viability was detected using MTT assay. It was determined that glipizide exposure on any of these 3 cell types has no significant impact on cell proliferation at all tested dosages (Figures 5A-C).

In view of the effect of glipizide on tumor cell proliferation and angiogenesis in CAM and YSM, we further determined the effect of glipizide on human umbilical vein endothelial cells (HUVEC) tube formation *in vitro*. We found that glipizide, even at the highest concentration (256 μM) tested, did not appreciably interfere with HUVEC growth in culture (Figure 5D). Cell migration, mediated by chemoattractants produced by vascular endothelial cells, is a hallmark of angiogenesis. In the CAM & YSM experiments, we observed that glipizide impaired angiogenesis. Hence, we examined HUVEC migration inside Boyden Chambers. We found that glipizide significantly attenuated HUVEC migration in a dose-dependent manner (Figure 5E). We also performed tube formation assay to determine the actions of glipizide on capillary formation flowing the introduction of HUVECs to monolayered Matrigel. As expected, glipizide-, but not DMSO-, treated HUVECs were inhibited from developing spider-like microvascular capillaries in a dose-dependent manner (Figures 5F and 5G).

### Glipizide inhibits angiogenesis through induction of vascular NPRA expression

**Figure 6:**
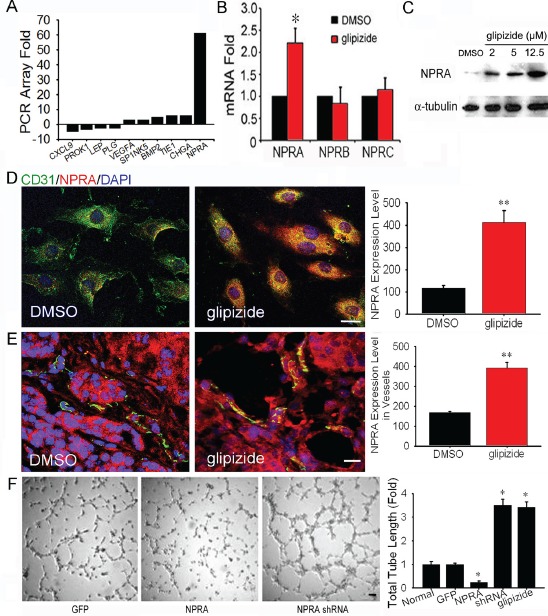
Glipizide inhibits angiogenesis through induction of NPRA expression (A) qRT-PCR array of genes associated with angiogenesis revealed NPRA expression was up-regulated 61-fold in HUVEC cells following glipizide treatment. (B-D) qRT-PCR (B), western blotting (C) immunofluorescent staining (D) were performed to confirm the microarray results. (E) Immunofluorescent staining revealed CD31 (endothelial cell marker) and NPRA was strongly expressed in the blood vessels of the spontaneous formed tumor tissues of MMTV-PyMT mice. (F) HUVEC cells transfected with GFP plasmid, full length human NPRA cDNA or NPRA shRNA. The effects of NPRA overexpression and silencing on vascular tube formation were examined 24 h after electroporation. The full length NPRA cDNA or NPRA shRNA induced NPRA over-expression or silencing in HUVEC cells shows silencing NPRA significantly increased the ability of HUVEC cells to form tubular structures. Furthermore, the ability of NPRA silencing HUVEC cells to form tubular structures did not change after glipizide treatment. * *p < 0.05*, ** *p < 0.01*. Scale bars = 20 μm in D and E and 100 μm in F.

We performed qRT-PCR array analysis for angiogenesis associated genes, using RNAs isolated from DMSO- and glipizide-treated HUVECs. We found that glipizide significantly induced NPRA expression amongst 90 known angiogenic related genes (Figure 6A). Interestingly, glipizide induces NPRA, but not NPRB and C, expression (Figure 6B). To confirm this finding, cultured HUVECs were immunofluorescently stained with NPRA and CD31 antibodies. Western blotting confirmed NPRA expression was increased in HUVEC treated with glipizide and the intensity of staining increased in a dose-dependent manner (Figure 6C). The immunofluorescent staining (Figure 6D and Supplemental Figure S2) also confirmed such observations. We examined whether glipizide could also up-regulate NPRA expression in the tumor endothelial cells of MMTV-PyMT mice. As expected, treatment of MMTV-PyMT mice with glipizide but not with DMSO, induced NPRA expression in CD31^+^ endothelial cells within solid breast tumors (Figure 6E). The NPRA staining was unevenly distributed along the tumor-induced vasculature, attesting to the ability of glipizide to act *in vivo*.

We assessed the importance of NPRA in angiogenesis by examining whether silencing NPRA can promote tube formation by HUVECs while ectopic expression of NPRA produces an inverse effect. We acquired plasmids carrying NPRA short-hairpin RNAs (shRNAs) and full-length NPRA cDNA. It has demonstrated that after silencing NPRA expression, the HUVECs increased microvascular tube formation while over-expression of NPRA inhibited tube formation (Figure 6F and Supplemental Figure S3). Furthermore, glipizide did not influence HUVECs tube formation after NPRA was silenced (Figure 6F). These results are consistent with previous finding that NPRA activation increases endothelial permeability [21,22], demonstrating the functional significance of NPRA in modulating vascular homeostasis.

## DISCUSSION

In this study, we report a significant finding that glipizide, a widely used antidiabetic drug, can potently inhibit angiogenesis by up-regulating NPRA expression in the vascular endothelial cells. Consequently, this leads to a reduction in microvessel density which attenuates tumor growth and metastasis - as determined using xenograft mouse models with breast carcinoma 4T1 cells and spontaneous breast carcinoma MMTV-PyMT mice.

The embryonic CAM and YSM assays are well established methods used in biological and pharmaceutical research because they are relatively simple and inexpensive, but highly reproducible [23]. In our preliminary studies, we used CAM and YSM approaches to screen an FDA approved drug library consisting of 480 compounds. Utilizing this high-throughput screen, glipizide has been identified with potent inhibitory effects on blood vessel formation within CAM and YSM. As such, we speculated that glipizide might be able to suppress tumor growth and metastasis by inhibiting tumor-induced angiogenesis.

On the other hand, recent pharmacoepidemiological surveys show that the use of antidiabetic drugs may influence cancer risk in type 2 diabetes mellitus. It has been reported that metformin, a widely used antidiabetic drug, is related to a reduced cancer incidence by acting on the aging process [24] and can potentiate chemotherapy in combination with rapamycin [25]. Furthermore, metformin can prevent breast cancer, especially chemoresistant or trastuzumab-resistant breast cancer, by targeted killing of cancer stem cell (CSC)-like breast cancer cells [26, 27]. It was reported that the patient treatment with glipizide or the same class of analogues may be associated with a lower cancer mortality than that with metformin [28]. Monami *et al*. [29] found that glibenclamide users had greater cancer-related mortality than gliclazide users. They reported that gliclazide treatment reduced cancer risk compared with that of glibenclamide [30]. It was also found that the diabetic patients treated with glipizide and metformin have lower risk to get cancer [31]. However, the relevant mechanism for such outcomes was unraveled. Our findings that glipizide attenuates angiogenesis, tumor growth and metastasis of inoculated mouse breast cancer 4T1 cells and MMTV-PyMT-driven breast carcinogenesis have provided direct evidence to support the cancer preventive and therapeutic effects of glipizide. Furthermore, we revealed the hypoglycemia developed in the mice treated with glipizide and glimepiride. However, there was no significant difference of the volume and weight between glimepiride and DMSO treatment, indicating the inhibition of tumor growth induced by glipizide treatment was not attributed to hypoglycemia. In addition, it was reported that the human plasma concentrations of glipizide are typically in the range of 50-300 ng/mL in diabetic patients receiving the drug therapeutically [32]. The glipizide dose of 100 μg per 20 g, which was administered to treat the tumor mice in our experiments, is consistent with the clinically used dose according to the body surface area. Therefore, the plasma concentrations of glipizide in tumor mice receiving the drug therapeutically are similar to those previously reported in patients, suggesting that glipizide may have an excellent safety profile as a potential anticancer drug for clinic use [33]. Given that bringing a new drug from discovery to the market is highly time-consuming and a costly process, repurposing of existing drugs represents an attractive strategy of cancer drug development [34]. To this end, our findings have demonstrated the promise of glipizide as a novel therapeutic that may benefit the cancer patients.

The tumor xenograft and transgenic mice experiments implied that angiogenesis might be involved in the inhibition of tumor growth and metastasis. We found that glipizide inhibited angiogenesis, tumor growth and metastasis in various tumor models. In order to discriminate the effects of glipizide on tumor cell proliferation and angiogenesis, we maintained breast cancer xenografts on chick CAM. The procedure allowed us to directly examine the correlation between tumor tissue and tumor angiogenesis in the presence of glipizide. Chick CAM assay has been extensively used in the past for this purpose [35]. We noticed that glipizide reduces the volume of the growing tumor in a dose-dependent manner. Likewise, the density of the vascular plexus in the tumor density also dramatically decreased. Our MTT assay revealed that glipizide has no significant effect against tumor cell proliferation. We also discovered that glipizide inhibited cell migration and the ability of these vascular endothelial cells to form tubular structures. These findings support glipizide inhibits tumor angiogenesis which results in tumor regression but not through suppressing tumor cell proliferation. Since angiogenesis is essential for tumor growth and metastasis, it has been widely trumpeted as a target for limiting tumor growth. Judah Folkman in 1971 was the first to suggest that tumor growth and metastasis were dependent on angiogenesis, and speculated that blocking angiogenesis could be an effective approach for inhibiting tumor growth and metastasis [36]. Given that antiangiogenic therapy presents advantages over other anticancer strategy, glipizide as a novel antiangiogenic inhibitor may be developed as a promising anticancer agent for clinical use.

To acquire a molecular insight into how glipizide suppresses angiogenesis, and better understand the relevant signaling pathways that glipizide exerts on the vascular endothelial cells, we performed qRT-PCR array analysis on glipizide-treated HUVECs for genes known to be associated with angiogenesis. We identified NPRA as the potential target and established that glipizide inhibited angiogenesis through the induction of NPRA expression in vascular endothelial cells. Sabrane *et al*. [22] reported that NPRA activation increases endothelial cell permeability, demonstrating that the receptor plays an important role in modulating vascular homeostasis. Meanwhile, it was recently reported that in NPRA-KO mice there was significantly lower angiogenic response compared with their wild-type counterparts [19]. NPRA has been shown to be expressed in cancer cells and proposed to be a potential prognostic marker and a target for cancer therapy [17,18]. Although Mallela *et al*. [19] concluded that NPRA signaling was involved in tumor growth through regulating angiogenesis, they only demonstrated that the numbers of CD31^+^ cells were decreased in the lung cancer sections of NPRA-KO mice compared with the wild-type mice by IHC staining. While these studies reported NPRA promoted cancer development, the relationship between NPRA and angiogenesis in the other kinds of tumors was not examined [19]. Here, our *in vitro* experiment showed that glipizide could not affect HUVEC tube formation when NPRA was silenced. For the first time, we demonstrated the role of glipizide and NPRA in tumor-induce angiogenesis.

Taken together, this study has shown that glipizide is capable of inhibiting tumor growth and metastasis in the tumor xenograft and transgenic mouse models. The inhibitory effect was principally achieved through the suppression of tumor-induced angiogenesis instead of cancer cell proliferation. Furthermore, NPRA has been identified as one of the potential antiangiogenic molecular targets for glipizide. While more research efforts are needed to elucidate the exact molecular mechanisms associated with its suppressive action on tumor growth, glipizide has demonstrated the great potential to be developed as a novel and promising anticancer drug for the treatment of various human cancers.

## MATERIALS AND METHODS

### Ethics Statement

All animal experiments were conducted according to relevant national and international guidelines. And this project was approved by the Medical Research Animal Ethics Committee of Guangdong Pharmaceutical University. When tumor volume exceeded 2cm^3^, mice were euthanized by cervical dislocation.

### Chemical reagents and cell lines

A chemical library containing 480 FDA-approved drugs or drug candidates were purchased from BIOMOL Company (BML-2840-0100). All the compounds were marked with a drug code (A0-A19; B0-B19; … X0-X19) and dissolved in dimethyl sulfoxide (DMSO, Amresco Company) to prepare 5 mg/mL stock solutions. Glipizide (cat. no. G117, Sigma, St Louis, MO, USA) was dissolved in DMSO to produce 5 mg/mL, 10 mg/mL and 20 mg/mL stock solutions for validation studies. Mouse breast cancer 4T1 cell line, human breast cancer MCF-7 cell line, human renal epithelial 293T cell line and human umbilical vein endothelial cell line were all purchased from the Institute of Biological Cells (Chinese academy of sciences, Shanghai, China).

### Chick embryos and mice

Fertilized white leghorn chicken eggs were obtained from the Avian Farm of South China Agriculture University (Guangzhou, China). The egg shells were cleaned using a 1% solution of geramine and then incubated in an egg incubator at 37.5 ^o^C and 50 - 60% humidity. Female 6 - 8 week old BALB/c mice were purchased from the animal center of Guangdong Province Medical School. MMTV-PyMT transgenic mice were obtained from the Jackson Memorial Laboratory. All the mice were weaned and tail-clipped at 4 weeks for genotyping by PCR. The primer pair used was specific for the MMTV-PyMT. All the mice were maintained in accordance with the NIH guidelines.

### Chick embryo chorioallantoic membrane (CAM) assay

The CAM assay was performed as previously described with a slight modification [37]. Briefly, fertilized eggs were incubated for 9 days and then the shell above the air chamber was cut using a dental saw to create a small window (10 × 10 mm^2^). After removal of the shell membrane with sterile forceps, 2, 4 and 8 μg of glipizide (20 μL) were applied directly to the air chamber of 9-day chick embryos. In the control group, the same concentration of DMSO in 20 μL of phosphate-buffered saline was applied. The eggs were then sealed with sterile medical tape and incubated at 37.8 °C and 60% humidity. After 48 h incubation, the CAM vasculature was photographed using a Canon camera. The images were analyzed using an image analysis program IPP 6.0 (Image Pro-Plus, version 6.0, Media Cybernetics) and the blood vessel density (percentage of blood vessel area over the whole area under microscopic field) was calculated. Each experiment was repeated twice with 10 eggs per experimental condition.

### Chick embryo yolk sac membrane (YSM) assay

Eggs were incubated for 3 days and then the chicken embryos were transferred into sterilized culture dishes. The individual yolk-sac vessels were oriented facing upward. Two silastic rings (inside diameter: 9.5 mm; outside diameter: 12 mm) labeled with red and black marker pens, respectively, were placed in symmetrical position over the yolk sac vessel area of the chicken embryos. The culture dishes were then covered tightly before returning to the incubator. 40 μL of glipizide (50, 100 and 200 μg/mL in gelatin) and DMSO were introduced into the rings of healthy embryos with well-developed vessels. Photographs of the vessels within the rings were captured at 0 h, 2 h and 24 h using an Image acquisition OPTPRO 2007 system. The images were quantitatively analyzed using an Image-Pro Plus 6.0 system, as described for the CAM assay.

### High-throughput screening for potential antiangiogenic drugs

A 20 μL aliquot of DMSO (control) or 50 μg/mL of one of 480 drugs (FDA-approved drug library) was added to each of the embryonic CAM or YSM membrane in the egg shell openings. The effect of all the tested compounds on angiogenesis was evaluated using the CAM and YSM assays.

### MCF-7 breast cancer assay on CAM

To evaluate the direct effect of glipizide on angiogenesis, MCF-7 breast cancer cells were cultured and maintained on the CAM of 10 day-old chick embryos [38]. Briefly, a 1 cm-diameter window was created in the shell of each 10 day-old fertilized egg. The CAM was exposed and a silastic ring was placed on top of the CAM. Then 40 μL of 0-200 μg/mL (2, 4 and 8 μg) glipizide was added to the center of the ring on day 12. On day 14, the eggs were cut carefully along the axis of the median line and the content was discarded. The gross morphology of each CAM was recorded using a stereomicroscope (SZX16 Olympus). The antiangiogenic effect of glipizide was evaluated by comparing the number of second- and third-order blood vessels with CAM treated with DMSO (control). The size (volume) of the breast tumors present on the CAM was calculated (length × width^2^ × 0.52). Statistical analysis of second- and third-order vessels was performed using 2-tailed Student’s t-test.

### 4T1 xenograft tumor model

4T1 mouse breast cancer cell line was injected subcutaneously into the second right mammary fat pad area of 6-8-week-old BALB/c mice. After inoculation, the mice were then randomly divided into glipizide, glimepiride (cat. no. G2295, Sigma) or DMSO (control) treatment. DMSO, glimepiride (4 mg/kg) or glipizide (5 mg/kg, according to clinically used dosage) was injected intraperitoneally into the 4T1 xenograft mice every day since the 4T1 cells were inoculated for six days. And the blood glucose levels were detected by blood glucose meter. The tumor volume was measured using a vernier caliper every three days and calculated using the formula: Volume = length × width^2^ × 0.52. The mice were treated with glipizide, glimepiride or DMSO for up to 20 days. The mice were then sacrificed and the tumors harvested for weighing and histological analysis.

### Metastasis assays

1 × 10^5^ 4T1 cells were injected into the tail vein of BALB/c mice followed by treatment with DMSO, glimepiride (4 mg/kg) or glipizide (5 mg/kg) on the same day of inoculation for 3 weeks. After 3 weeks, the mice were sacrificed, and their brains, lungs, livers, and kidneys were collected for analysis. The tissue samples were analyzed for the presence of metastatic foci and histology.

### MMTV-PyMT transgenic mice

Nine-week-old MMTV-PyMT mice were intraperitoneally injected with DMSO or glipizide (5 mg/kg) for 20 days. These mice spontaneous develop breast carcinoma. The tumors were measured every three days and the tumor volume was calculated: length × width^2^ × 0.52. After 20 days, the mice were sacrificed and their tumors and lungs were collected for weighing, histological analysis and pulmonary metastatic foci counting.

### Immunohistological and immunofluorescent staining

Immunohistochemistry and immunofluorescent staining were performed on 3-μm and 6-μm sections, respectively. Before immunohistochemical staining, transgenic and 4T1 xenograft mice were intraperitoneally injected with 100 mg/kg of 5-bromo-2′-deoxyuridine (BrdU; cat. no. B5002, Sigma). Briefly, the sections were dewaxed, hydrated and incubated anti-CD31 (1:100 dilution, cat. no. sc-1506, Santa Cruz, CA, USA), anti-BrdU (1:100 dilution, cat. no. sc-32323, Santa Cruz, CA, USA) and anti-NPRA (1:100 dilution, cat. no. sc-25485, Santa Cruz) primary antibodies, overnight at 4 ^o^C. The binding of the primary antibodies to cells/tissues was detected using the appropriate fluorescently labeled secondary antibody or HRP-conjugated secondary antibody plus DAB. All sections were the counterstained with hematoxylin or DAPI. The density of microvessels was quantified by counting the number of CD31^+^ vessels in a 200× field [39]. For BrdU quantitation, fields were randomly selected in a 400× field and the number of BrdU^+^ cells were counted as a percentage of the total cells per field. The NPRA expression levels were quantified using an image analysis program IPP 6.0 (Image Pro-Plus, version 6.0, Media Cybernetics) in a 400 × field and evaluated by two experimenters.

### In situ hybridization

*In situ* hybridization (ISH) was performed to determine a vascular endothelial growth factor receptor-2 (VEGFR-2) [38]. The PCR product (365 bp) was cloned into T-vector with T3 and T7 promoter and the riboprobe was labeled with dNTP containing Bio-dUTP through transcribing T3 promoter transcriptase. After the tumor tissue sections were treated by proteinase K (0.2 mg/mL), the probe was incubated overnight at 37°C. Then the Digxion-Avidin was used to bind the probe and at last the tissue sections were stained with DAB. The PCR primer is as follows: AAGAGGATTCGGGCCTCTCT, CCCTGACTGGTAGCCACTTG.

### MTT assay

MTT assay was performed to determine the effect of glipizide on cell proliferation. Briefly, cells were introduced into 96 well plates and then treated with various dose of glipizide. After incubation for 48-72 h, 10 μL of MTT reagent was added to each well. The plates were incubated at 37 ºC for 4 h and then the supernatant removed. The resultant formazan crystals were dissolved using 150 μL of DMSO (Sigma). The absorption was read at 570 nm through a spectrophotometer. The assay was performed in triplicate.

### Tube formation assay

The effect of glipizide on vascular tube formation was determined using HUVEC cells. The cells (1.5 × 10^5^ cells/mL) in suspended EBM were uniformly spread onto growth factor-reduced matrigel in 96-well chamber. After the plates were incubated for 5 h at 37 ºC, the presence of vascular tubes was photographed using a phase contrast inverted microscope. The length of the tubes was measured using image analysis software.

### Cell migration assay

Boyden Chamber Transwells were used to evaluate cell migration ability. HUVEC cells were maintained in serum-free media for 5 h. Cell migration was examined as a standard protocol. Briefly, a porous polycarbonate membrane (8-μm pore diameter; cat. no. PFB8, Neuro Probe, ) was coated with 1% gelatin (Sigma) for 1 h and sterilized. HUVEC cells were harvested and suspended in serum-free EBM medium containing different concentrations of glipizide. 50 μL of cells (2.5 × 10^4^ cells) were introduced to the upper chamber of the transwell and the lower chambers were added culture medium containing 20% FBS plus various growth factors (SDF1, HB-EGF, VEGF and HGFApoG2). The polycarbonate membrane was inserted between the upper and lower chambers. After the cells in the Boyden Chamber were incubated at 37 ºC for 12 h, the cells were then removed from the upper side of the membrane by using a cotton swab. And then the cells were stained with 1% crystal violet for 3 h after fixed in 4% paraformaldehyde overnight. The cells were counted using a microscope at 4 × magnification.

### Quantitative real-time PCR

Total RNA was extracted from HUVEC cells that have been treated with glipizide or DMSO (control). The PCR array was performed using a TaqMan-based qPCR assay kit (cat. no. PAMM-024A; SA Biosciences). An ABI PRISM 7000HT Sequence Detection System (Applied Biosystems) was used for all qPCR analysis. Triplicate qPCR reactions were performed for each of the samples analyzed.

### Western blotting

HUVECs cells were harvested and suspended in lysis buffer to extract whole-cell protein. 60 μg of total protein was then separated by SDS-PAGE (10% polyacrylamide gel) and transferred onto nitrocellulose membranes. Antibody against NPRA was purchased from Santa Cruz, and the immunoblotting was performed according to manufacturer’s instructions.

### Over-expression and silencing NPRA

Full length NPRA cDNA or short hairpin RNA (shRNA) constructs that targeted the NPRA transcript were purchased from Open Biosystems. Cells were transfected with these constructs along with plasmid carrying the green fluorescence protein (GFP) marker. The efficacy of these constructs in over-expressing and silencing NPRA was validated by immunoblotting using NPRA antibody.

### Statistical analysis

Data analyses and drawing of statistical charts were performed using a Graphpad Prism 5 software package (Graphpad Software, CA). The results were statistically analyzed using a two-tailed Student’s t-test, as the prerequisites (independence and normal distribution) were satisfied. Data gathered from immunohistochemistry, tumor volumes and tissue weights of compared between glipizide-treated and control specimens. Differences between the groups were considered significant at *p< 0.05*.

### Authors’ Contributions

Conception and design: C. Qi, Q. Zhou, Y. Yang, B. Li, Y. Ye, J. Li, Y. Ding, H. Wang, J. Wang, X. He, X. Yang, L. Wang.

Development of methodology: C. Qi, Q. Zhou, Y. Yang, B. Li, Y. Ye, J. Li, Y. Ding, H. Wang, J. Wang, X. He, X. Yang, L. Wang.

Acquisition of data (provided animals, acquired and managed patients, provided facilities, etc.): C. Qi, Q. Zhou, Y. Yang, B. Li, Y. Ye, J. Li, Y. Ding, H. Wang, J. Wang, X. He, X. Yang.

Analysis and interpretation of data (e.g., statistical analysis, biostatistics, computational analysis): C. Qi, Q. Zhou, Y. Yang, B. Li, Y. Ye, J. Li, Y. Ding, H. Wang, J. Wang, X. He, X. Yang, L. Wang.

Writing, review, and/or revision of the manuscript: C. Qi, L. Cao, K.K.H. Lee, J. Zhou, X. Yang, L. Wang.

Administrative, technical, or material support (i.e., reporting or organizing data, constructing databases): C. Qi, Q. Zhou, Y. Yang, B. Li, Y. Ye, J. Li, Y. Ding, H. Wang, J. Wang, X. He, X. Yang, L. Wang.

Study supervision: C. Qi, Q. Zhang, T. Lan, W. Li, X. Song, X. Yang, L.Wang.

## SUPPLEMENTARY FIGURES AND TABLES


