# Supplementary material for: Glipizide, an antidiabetic drug, suppresses tumor growth and metastasis by inhibiting angiogenesis
Source: Oncotarget. 2014 Sep 16;5(20):9966–79. doi: 10.18632/oncotarget.2483 (PMC4259451; doi:10.18632/oncotarget.2483)
Supplement: Supplementary file 1 [file oncotarget-05-9966-s001.pdf]

Glipizide, an antidiabetic drug, suppresses tumor growth and metastasis by inhibiting angiogenesis

Supplementary Material

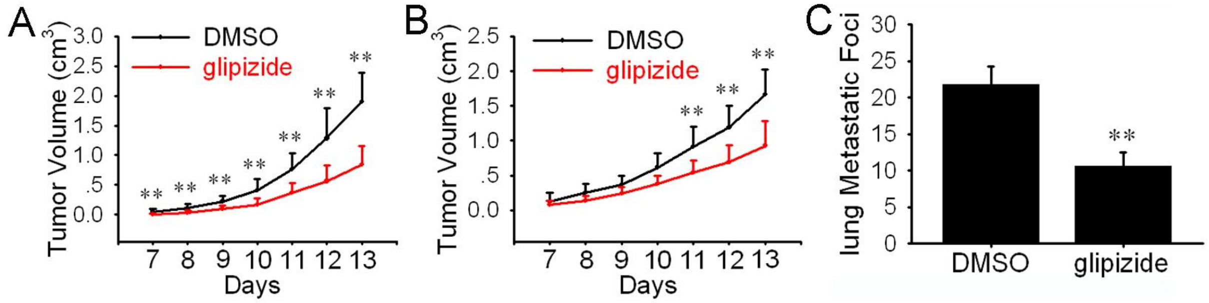

Figure S1: Glipizide inhibits tumor growth and metastasis of melanoma B16 cells. B16 cells were injected into the right armpit and when tumors were palpable on day 7, they were injected with glipizide (5 mg/kg) or DMSO (control) once every day. A and B, The mice were treated on the first or the seventh day of the B16 cells inoculation. The volume of the melanoma was measured every day, indicating that the tumor volume significantly decreased after glipizide treatment. C, B16 cells were intravenously injected into the mice. Incidence of lung metastasis enumerating from the breast tumors after glipizide and DMSO treatments. Results are shown as mean ± S.D. Values of three independent experiments. *$p<0.05$, and **$p<0.01$.

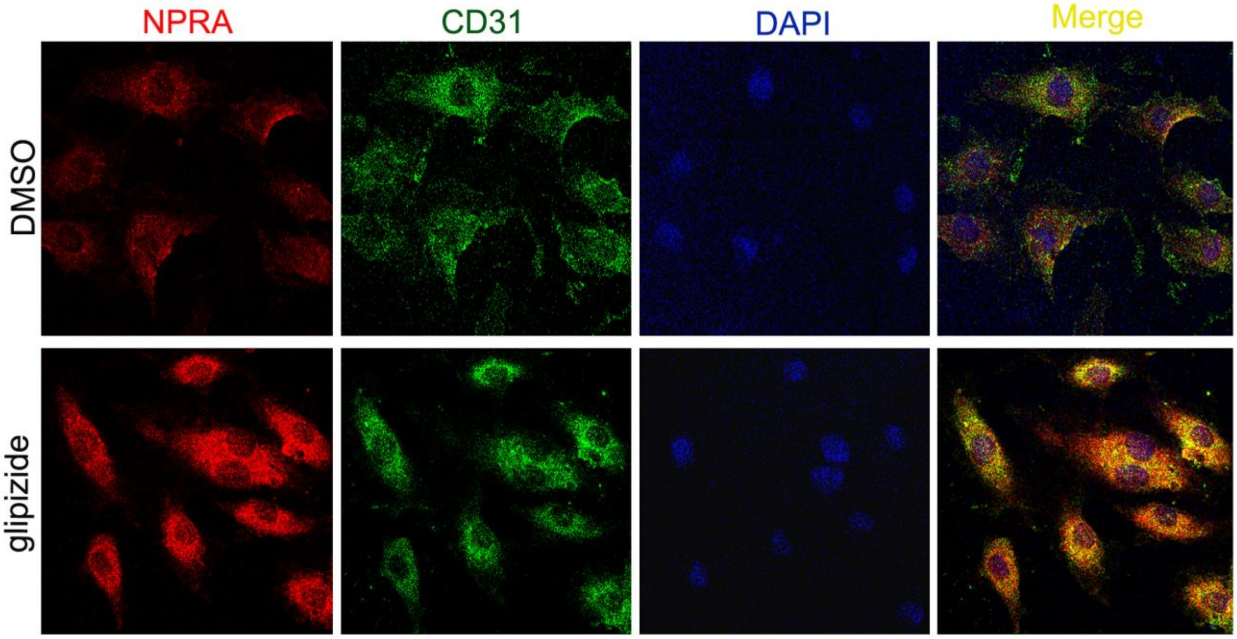

Figure S2. Glipizide induces NPRA expression in HUVEC cells. Immunofluorescent staining revealed CD31 and NPRA was strongly expressed in HUVEC cells treated with glipizide.

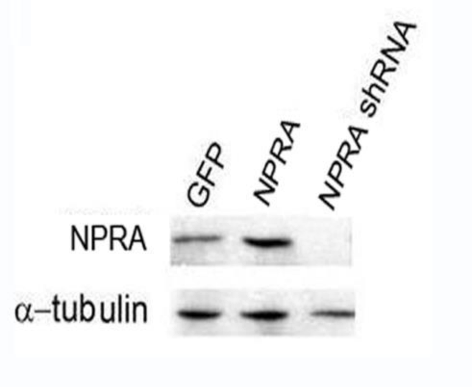

Figure S3. The full length NPRA cDNA or NPRA shRNA induced NPRA over-expression or silencing in HUVEC cells. HUVEC cells were electroporated with GFP plasmid, full length human NPRA cDNA or NPRA shRNA. The expression of NPRA was examined by western blotting 24 h after electroporation.

Table S1: High-throughput screening identified glipizide as a potent antiangiogenic drug. DMSO (control) and the small molecules dissolved in DMSO were introduced on chick embryo CAM. Preliminarily screen identified 27 compounds that could potently inhibit angiogenesis. The same dosage of the 27 compounds was administered in YSM assay which narrowed down 1 (glipizide) out of the 27 compounds with antiangiogenic effects. Glipizide as the most potent compound was selected for further studies.

Methods	Potentially antiangiogenic compounds identified
CAM	P1A10, P2E10, P1C9, P3H4, P5G6, P1C5, P4E7, P1B2, P1B6, P1B10, P1C6, P1D3, P1D4, P1E4, P1G2, P1H2, P1H6, P2B5, P2B6, P2E11, **P3B6**, P3H2, P5D7, P5D8, P5G4, P5H8, P5H11
YSM	**P3B6 (glipizide)**

Table S2: Glipizide and glimepiride lower blood glucose levels in BALB/c mice. The postprandial blood glucose levels in BABL/c mice treated with glipizide and glimepiride were reduced 30 min later in comparison with the DMSO treatment (control). Furthermore, the postprandial blood glucose levels returned to normal on 12 h after the treatment of glipizide and glimepiride, respectively.

Groups	Postprandial blood glucose		
	0 h	0.5 h	12 h
DMSO	5.84 ± 0.34	6.12 ± 0.29	6.06 ± 0.62
glimepiride	5.52 ± 0.60	4.38 ± 0.49**	5.96 ± 0.83
glipizide	5.66 ± 0.34	4.54 ± 0.65**	5.98 ± 0.45

Table S3: The blood glucose levels were reduced in MMTV-PyMT mice treated with glipizide. Compared with the DMSO treatment, the postprandial blood glucose levels in the MMTV-PyMT mice were significantly decreased at 30 min and returned to normal 12 h later.

Groups	Postprandial blood glucose		
	0 h	0.5 h	12 h
DMSO	10.08 ± 0.98	11.04 ± 0.67	10.12 ± 0.88
glipizide	10.3 ± 0.91	6.5 ± 0.82**	9.83 ± 0.74
